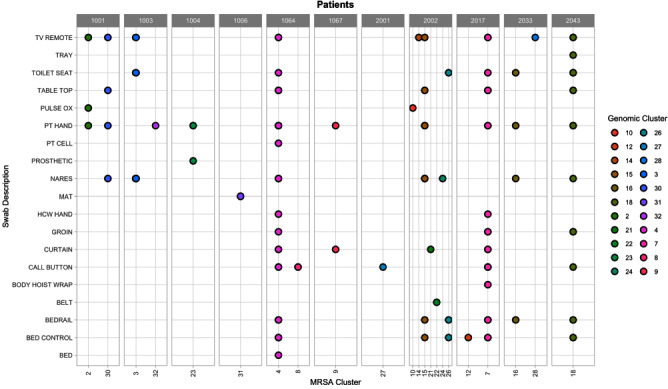# Capturing MRSA Diversity by Integrating Genomic and Epidemiological Data of Patients and their Spaces

**DOI:** 10.1017/ash.2024.116

**Published:** 2024-09-16

**Authors:** Tasmine Clement, Kristen Panson, Marco Cassone, Ali Mohammed Pirani, Evan Snitkin, Lona Mody

**Affiliations:** University of Michigan; VA Ann Arbor Healthcare System

## Abstract

**Background:** Methicillin-resistant S. aureus (MRSA) is known to cause frequent and severe infections in community living centers, potentially resulting in significant mortality for elderly patients. More research is needed to understand how to utilize genomic and epidemiological data to understand characteristics that may lead to increased transmission. We hypothesized that combining genomic and epidemiological information to sample patients and their environments during long-term stays, we will be able to capture a diverse set of MRSA strains. **Method:** This work included genome sequencing of patient and environmental samples from 11 patients within the VA Ann Arbor Healthcare System from May 4, 2021- November 16, 2022. All 11 patients tested positive for MRSA during their stay (mean days = 31). Patient and environmental samples were taken throughout their stays, screened for MRSA, and whole-genome sequenced. Single nucleotide variants (SNVs) were identified by mapping reads and calling variants against strain-specific reference genomes. We used ape v5.6-2 in R v4.2.2 to analyze and infer evolution, acquisition, and transmission events based on pairwise SNV distances. Genomic clusters were determined using stats v3.6.2, with a SNV distance threshold of 20. **Result:** Samples that were collected from patient bodily sites were able to reveal 20 distinct genomic clusters of MRSA (patient hands: n = 10, nares: n = 7, groin: n = 3). Environmental samples from patient environments also revealed distinct genomic MRSA clusters (tv remote: n = 9, toilet seat and bed rail: n = 6, table top, bed control, and call button: n = 5, bed curtain: n = 4, pulse ox = 2, cell phone, tray, pulse ox, mat, body hoist wrap, and bed: n = 1). **Conclusion:** The identification of various genomic clusters from patients and their environmental reservoirs suggests intrahost variation that can only be captured by using a more holistic approach of integrating epidemiology and genomic sequencing. Developing studies that incorporate genomic data, various environmental sources, and multiple isolates over time within community living centers can increase our understanding of strains that are more likely to transmit, survive on living and non-living surfaces and therefore lead to improved recommendation for infection prevention interventions and drivers of endemicity.

**Disclosure:** Lona Mody: NIH, VA, CDC, Kahn Foundation; Honoraria: UpToDate; Contracted Research: Nano-Vibronix

**Figure:**